# A grey area: how does image hue affect unfamiliar face matching?

**DOI:** 10.1186/s41235-019-0174-3

**Published:** 2019-07-22

**Authors:** Anna K. Bobak, Viktoria R. Mileva, Peter J. B. Hancock

**Affiliations:** Psychology, Faculty of Natural Sciences, University of Stirling, Cottrell Building, Stirling, FK9 4LA UK

**Keywords:** Face matching, Unfamiliar faces, ID checks, National security, Face processing

## Abstract

The role of image colour in face identification has received little attention in research despite the importance of identifying people from photographs in identity documents (IDs). Here, in two experiments, we investigated whether colour congruency of two photographs, shown side by side, affects face-matching accuracy. Participants were presented with two images from the Models Face Matching Test (experiment 1) and a newly devised matching task incorporating female faces (experiment 2) and asked to decide whether they show the same person or two different people. The photographs were either both in colour, both in grayscale, or mixed (one in grayscale and one in colour). Participants were more likely to accept a pair of images as a “match”, i.e. same person, in the mixed condition, regardless of whether the identity of the pair was the same or not. This demonstrates a clear shift in bias between “congruent” colour conditions and the mixed trials. In addition, there was a small decline in accuracy in the mixed condition, relative to when the images were presented in colour. Our study provides the first evidence that the hue of document photographs matters for face-matching performance. This finding has important implications for the design and regulation of photographic ID worldwide.

**Electronic supplementary material:**

The online version of this article (10.1186/s41235-019-0174-3) contains supplementary material, which is available to authorized users.

## Significance statement

Photographic documents, such as national identity cards, driving licences, and passports are the most common means of verifying an individual’s identity. This is despite most of the research suggesting that unfamiliar face matching is difficult and error prone. Much attention in the literature has been devoted to factors influencing face matching, such as image quality, time between the taking of photographs, and the presence of paraphernalia, such as glasses. However, no work has considered the influence of the colour of photograph on the accuracy of face matching, and current identity documents (IDs) are often printed in grayscale, e.g., the European Union (EU) driving licence or Polish and Canadian passports. The findings of this paper highlight the potential pitfall of using grayscale images in IDs. People are more inclined to accept a pair of images as a match when one is grayscale and one is in colour. This detrimental effect is particularly important in the mismatched trials, i.e. when the two images present two different people. While it is unclear whether this effect persists in trained or highly skilled individuals (e.g., passport officers), our participants were sampled from a population that often works in service sector industries where routine ID inspections are commonplace. We call on the policy makers to re-think image colouration in photographic identity documents.

## Background

From passport checks to buying age-restricted items, photographic identity documents (IDs) are the most commonly used proof of one’s identity. Although passport control increasingly relies on automated technology, when the identity is in question, or when the passport holder is a minor, human observers make the final decision.

Research has repeatedly shown that face matching is a challenging task and even motivated and trained individuals make a considerable number of mistakes (Kemp, Towell, & Pike, 1997; White, Kemp, Jenkins, Matheson, & Burton, 2014), often independently of experience (White, Kemp, Jenkins, Matheson, and Burton, 2014; Wirth & Carbon, 2017). In their seminal study, Kemp et al. (1997) examined the accuracy of experienced cashiers in detecting fraudulent IDs. They found that despite a financial incentive to do well, cashiers accepted approximately 35% of foil ID cards even when the appearance of the card bearer did not resemble that of the foil depicted on the document’s image. Under optimal laboratory conditions, when photographs are taken on the same day, participants sampled opportunistically from the general population make between 11% and 20% of mistakes in a matching task (Burton, White, & McNeill, 2010). In real-life settings, these optimal conditions are rarely preserved. With a typical passport document valid for ten years, factors such as age (e.g., White, Phillips, Hahn, Hill, & O’Toole, 2015), hairstyle changes (Ellis, Shepherd & Davies, 1979), wearing glasses (Kramer & Ritchie, 2016), and general within-person appearance idiosyncrasies (Ritchie & Burton, 2017) can all be detrimental to face-matching accuracy.

To address this issue, a number of studies have concentrated on the individual differences in face matching and the ways to improve photographic ID by, for instance, providing multiple images of the same person (Dowsett, Sandford, & Burton, 2016), restricting the viewing to internal features (Kemp, Caon, Howard, & Brooks, 2016), face-matching training (Alenezi & Bindemann, 2013; Dowsett & Burton, 2015; Moore & Johnston, 2013; White, Kemp, Jenkins, & Burton, 2014), and by giving specific instructions on which features to focus on (Megreya & Bindemann, 2018).

With the limited success of training regimes (c.f., Megreya & Bindemann, 2018) and few effective ways of improving ID documents for human observers, several studies proposed that selecting individuals from the high end of the face processing ability spectrum would be the best strategy for improving operational accuracy while more adequate training methods are developed (Bobak, Dowsett, & Bate, 2016; Bobak, Hancock, & Bate, 2015; Robertson, Noyes, Dowsett, Jenkins, & Burton, 2016). Indeed, so-called super-recognisers have been found to outperform typical perceivers on standard face-matching tasks both as a group and at the individual level (Bobak et al., 2016; Robertson et al., 2016) with some performing on par with or better than the leading computer algorithms (Phillips et al., 2018). However, the possibility of employing super-recognisers for all possible face-matching scenarios (i.e., border control and selling age-restricted items in stores) is unlikely. Therefore, most face-matching tasks will continue to be problematic.

While experimental work on face matching has typically concentrated on *person properties -* the variability in individual appearance such as that caused by facial expression, hairstyle, pose, age, or paraphernalia - in face-matching accuracy, considerably less research has examined *image properties* (i.e., changes that can be applied after images have been taken) and their effect on face processing. One such image property is colour, previously shown to be relevant for face recognition (Yip & Sinha, 2002), face detection (Bindemann & Burton, 2009), gender classification (Nestor & Tarr, 2008), and non-face object recognition (Bramão, Reis, Petersson, & Faísca, 2011). Kemp, Pike, White, and Musselman (1996) showed that completely inverting the hue, such that a typical face appears in shades of blue, had almost no effect on the recognition of familiar faces but did affect recognition of previously unfamiliar faces. Yip and Sinha (2002) showed that colour information does matter for face recognition when availability of other cues is diminished, for instance when faces are blurred, but not when the images are of high quality. This is due to colour information facilitating low-level analysis and segmenting features within a face (such as separating the mouth contour or the hairline), rather than aiding identification directly (but see Abudarham & Yovel, 2016; Bindemann & Burton, 2009). However, Abudarham and Yovel (2016) identified several critical features, such as hair and eye colour, that are invariant across changes in one’s appearance and are pertinent to recognising one’s identity. Changing these features appears to considerably alter the perception of identity, while variations in other features do not. For instance, chin shape was defined as a non-critical feature that differs depending on rigid and non-rigid face motion, but eye colour and hair colour remain the same, providing they are not disguised deliberately with coloured contact lenses or hair dye.

**Fig. 1:**
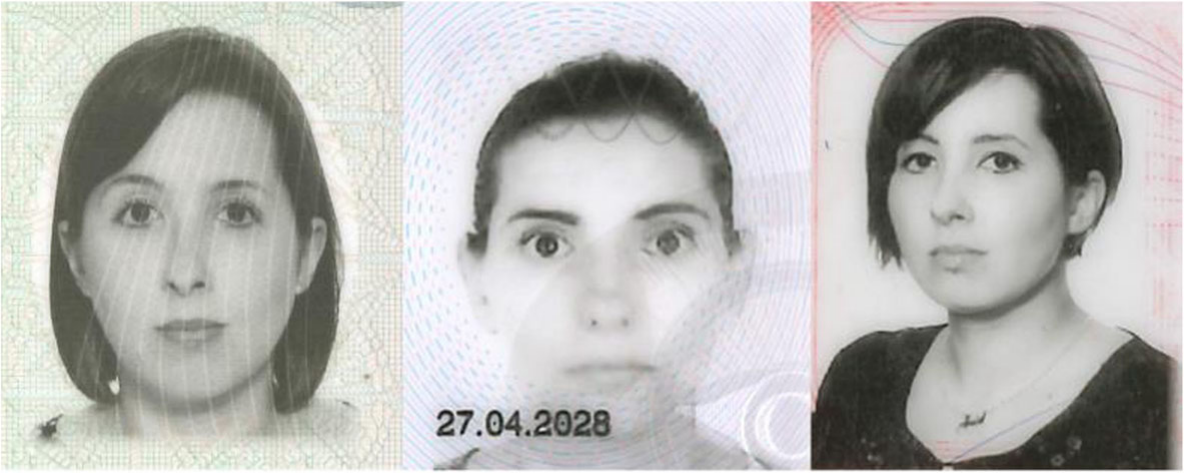
Examples of photographs (from left to right) from a Polish passport, a UK driving license, and a Polish identity card. Persons depicted in these images have given permission for them to be used as illustration

It is thus plausible that colour is an important factor not only in face recognition, or detection, but also in face matching, yet one of the most commonly used tests to assess the face matching ability, the Glasgow Face Matching Test (Burton et al., 2010) is administered using grayscale images, while other tasks, such as the Model Face Matching Test (MFMT) (Dowsett & Burton, 2015), or the new Kent Matching Test (Fysh & Bindemann, 2017) utilise colour photographs. It is unclear what effect image colour incongruence may elicit on face-matching performance. This is important, because in real-life situations, it is common for a grayscale ID photograph to be compared with an individual in front of the person performing the check. For instance, EU driving licences, Polish national identity cards that are valid for international air travel within the EU, and Polish and Republic of Ireland passports contain grayscale photographs (for examples see Fig. 1). Other countries such as Canada allow applicants to submit either grayscale or coloured photographs for their passports. These documents are used for identity verification at airports and when buying age-restricted items. Thus, if image hue influences face-matching performance, this could have important implications for the design of photographic ID.

In this study, we investigated whether image colour affects accuracy in the matching of photographs. We used unconstrained images from the well-established MFMT (Dowsett & Burton, 2015) and a newly designed face-matching task capturing the natural variability in people’s appearance. This is important, because in real-world situations people vary in their everyday appearance and many IDs do not have to adhere to strict passport-like image capture guidelines. We tested participants under three conditions: “colour”, “grayscale”, and “mixed” (where one image was presented in colour and one in grayscale). The addition of the mixed trials is the main advancement of this study on those previously reported in the literature and is of importance from both theoretical and applied perspectives. We hypothesised that, if colour facilitates low-level analysis, it is possible that grayscale images and/or hue incongruency between photographs may disrupt this process leading to a decrease in overall accuracy in these conditions, relative to when both images are presented in colour. Additionally, if hair and eye colour are critical features that individuals use for recognising unfamiliar individuals (Abudarham & Yovel, 2016), we would expect decreased performance in “mixed” and grayscale conditions. However, if colour is a general diagnostic (i.e. it is helpful for extracting a robust representation of one’s face by integrating hue, shading, and fine-grained featural information) for one’s identity from which one can generalise to other instances of the same identity, one clear and high-quality image may be enough to extract identity information sufficient to compare this identity to a second picture in a “mixed” matching trial. We would then merely expect reduced performance in the grayscale trials.

## Experiment 1

### Method

#### Participants

A total of 42 students (30 female; age, mean (M) = 20, SD = 3.5; all with self-reported normal or corrected-to-normal vision) at a university in the UK took part in the study on a voluntary basis and without reimbursement. The study was approved by the General University Ethics Panel and was carried out in accordance with the recommendations of the World Medical Association Declaration of Helsinki. Sample size was determined based on previous research (e.g., Kramer & Ritchie, 2016) and our stopping point was set for the pre-determined participant number.

#### Materials

**Fig. 2:**
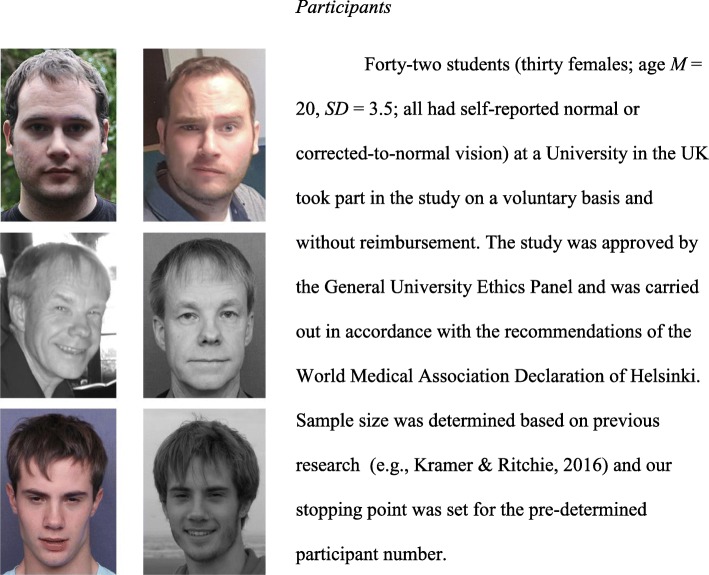
Images showing the three conditions for three different identities. Top row shows “colour”, middle row shows “grayscale”, and bottom row shows “mixed” conditions. All pairs are same-image pairs. Copyright restrictions prevented the publication of the original photographs. Individuals depicted in this figure did not appear in the experiment. All have given permission for their images to be reproduced

Our materials consisted of a total of 90 MFMT trials: 45 matched and 45 mismatched trials divided into three sets of 30 trials (15 matched and 15 mismatched per set). All three sets were of equal difficulty (this baseline average accuracy for each set was determined by pilot testing in Dowsett & Burton, 2015). In this study, we called these three sets of 30 face pairs A, B, and C. All images measured 300 (width (W)) × 420 (height (H)) pixels, did not contain visible jewellery, but were not cropped of hair or clothing to mimic natural conditions under which face matching would occur (Fig. 2). We created three variations of every pair: (1) *colour* condition as per the original study, (2) *grayscale* condition where all were presented in black and white, and (3) *mixed* condition, where one image of each pair was presented in colour and one in grayscale. Images were converted from colour to grayscale using IrfanView software (http://www.irfanview.com/).

#### Procedure and apparatus

Each participant saw all 90 pairs. The colour condition in which they saw each set was counterbalanced, i.e. some participants saw set A in colour, some saw it in the mixed, and others in the grayscale condition etc. All participants saw all three colour conditions (within-subjects design) displayed randomly (not blocked) to mimic the natural environment in which those checking identity documents may operate (see Fig. 2 for examples of face pairs). In the mixed condition pairs, the grayscale images appeared equally often on each side of the screen.

On each of the 90 trials, the pairs of images were presented side by side, one to the left and one to the right of the centre of the screen. The viewing distance was not fixed. Participants were instructed to decide whether two images presented on screen were of the same person, or two different people and respond with the “s” key for “same” and “k” key for “different”. These response buttons remained the same throughout the experiment for each participant. There was no time restriction placed on participants. Testing took part in dimly lit cubicles using 19 in. monitors running 1280 × 1024 pixels resolution, and refresh rate 60 Hz.

### Results

All participants’ data were used in the analyses. Accuracy was analysed separately for matched and mismatched trials due to the weak correlation between performance on matched and mismatched trials as reported in the literature, which suggests that these trials represent distinct processes (Megreya & Burton, 2007).

For matched trials, percentage correct was analysed using one-way within-subjects analysis of variance (ANOVA) with three levels (colour, grayscale, and mixed). There was a significant main effect of image hue, *F*(2,82) = 9.96, *p* < .001, η^2^_p_ = 0.19. Follow-up pairwise comparisons (Bonferroni corrected) showed that participants were more accurate in the “colour” and “mixed” conditions than in the “grayscale” condition, *p* = .045, *d* = 0.40 (95% CI 0.11, 0.72) and *p* < .001, *d* = 0.77 (95% CI 0.44, 1.15), respectively (see Table 1 for a summary of means and SD). The mixed and colour conditions did not differ from each other: *p* = .279, *d* = 0.29 (95% CI − 0.04, 0.64).

**Table 1:** Average performance for all conditions (standard deviations are in parentheses)

Condition	“Match” accuracy (%)	“Mismatch” accuracy (%)	Overall accuracy (%)	Sensitivity (*d’*)	Criterion (*c*)
Colour	57.4 (20.1)	73.8 (19.7)	65.6 (11.0)	0.97 (0.70)	0.25 (0.52)
Grayscale	49.7 (19.0)	77.8 (17.6)	63.8 (10.4)	0.89 (0.65)	0.45 (0.49)
Mixed	62.5 (15.1)	61.7 (19.4)	62.1 (11.0)	0.70 (0.68)	0.00 (0.39)

Accuracy was also examined in mismatched trials, using within-subjects ANOVA with three hue levels. There was a significant main effect of condition, *F*(2,82) = 23.60, *p* < .001, η^2^_p_ = 0.365. Pairwise comparisons (Bonferroni corrected) revealed that performance was lower in the mixed condition than in colour and grayscale conditions, *p* < .001, *d* = 0.64 (95% CI 0.34, 0.98) and *p* < .001, *d* = 0.89 (95% CI 0.56, 1.28), respectively. Accuracy in grayscale and colour conditions did not differ, *p* = .073, *d* = 0.22 (95% CI 0.04, 0.41).

In keeping with other recent studies in the field of face matching, we also analysed signal detection measures to separate the effects of sensitivity and response bias on match and mismatch image trials. *d* prime was calculated by subtracting the *z* scores for false alarms (FA), i.e. when participants responded “same” in mismatched trials, from z scores when participants correctly identified two images as “same” in matched trials (hits, H). Response bias (*criterion c*) was calculated by taking a negative average of *z* scores for the H and FA responses (Macmillan & Creelman, 2004). On one-way within-subjects ANOVA of *d* prime scores there was a non-significant trend for hue condition, *F*(2,82) = 2.65, *p* = .077, η^2^_p_ = 0.06. However, the critical comparison is between the colour and mixed conditions and these differed significantly on analysis by paired *t* test, *t*(41) = 2.31, *p* = .028, *d* = 0.35 (95% CI 0.05, 0.66).

**Fig. 3:**
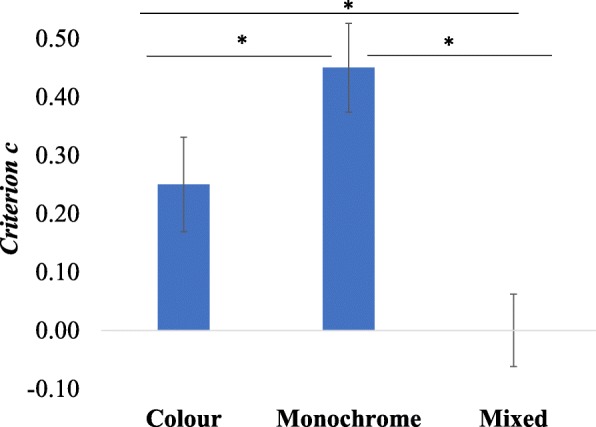
Differences in response bias across three hue conditions in experiment 1. Error bars represent the SEM

An analogous analysis of response bias, showed a highly significant main effect of condition *F*(2,82) = 29.24, *p* < .001, η^2^_p_ = 0.42, with a very large effect size. Follow-up comparisons (Bonferroni corrected) showed that participants had a significantly more conservative bias (they were more likely to reject a pair as mismatch) in the grayscale condition than in the colour: *p* = .002, *d* = 0.39 (95% CI 0.18, 0.60), and mixed conditions *p* < .001, *d* = 1.03 (95% CI 0.71, 1.41). Participants were also more likely to respond “different” in the colour condition, than in mixed condition*, p* < .001, *d* = 0.57 (95% CI 0.28, 0.90) (see Fig. 3). This reflects the matching data shown in Table 1: the mixed condition produces the highest match accuracy, at the cost of the worst mismatch accuracy: participants are simply more likely to declare a match than in the colour and grayscale conditions.

### Discussion

In experiment 1, we examined how image hue affects face-matching performance in a group of young British adults. While the overall accuracy did not differ between conditions (Table 1), when we examined *d* prime there was a trend towards individuals being better at discriminating faces (i.e. deciding whether they were the same or two different faces) when they were presented in colour relative to when the colour of images differed. Even more clearly, there were differences in how participants approached matched and mismatched trials depending on the colour congruency. In the colour and grayscale conditions, participants were significantly more biased to respond conservatively (i.e. that a pair was a mismatch). This was more pronounced for the grayscale pairs. This pattern of responses was not present in the mixed-hue condition where the accuracy was comparable in both matched and mismatched trials. More importantly, in mismatched trials, participants were significantly less accurate than in colour and grayscale conditions. This clear shift in bias (see Fig. 3) may be explained by the additional difference between the two images in each pair. That is, in colour and grayscale conditions each of the two side-by-side images only differed in the specific pictures displayed (see Fig. 2 top and middle rows). However, within the mixed condition, the two images differed in which pictures were displayed but also in the extra dimension of having one in colour and one in grayscale. This may have led participants to discount perceptions of a difference between the two images (in mismatch pairs), and to attribute those differences to the image hues, rather than differences in actual identities.

These biases were unexpected, and because we anticipated differences in performance to affect the mixed condition irrespective of the trial type, we sought to replicate this effect in another experiment with a more ecologically valid face set (Additional file 1).

## Experiment 2

### Method

#### Participants

A total of 52 psychology students (46 female; age *M* = 21, *SD* = 5.3 years; all with self-reported normal or corrected-to-normal vision) at a university in the UK took part in the study in exchange for credits required as a part of one of the modules. The study was approved by the General University Ethics Panel and was carried out in accordance with the recommendations of the World Medical Association Declaration of Helsinki. Our stopping point was again a pre-determined participant number.

#### Materials

We downloaded two photographs of 96 Polish, Swedish, and Croatian celebrities (half of whom were female). The images depicted people in different settings, so matching could not be completed based on background features, clothing, or paraphernalia. All images depicted faces with both eyes, nose and the mouth visible. For half of these identities, we downloaded images of celebrity foils matched on gender, approximate age, hair, and eye colour. All images measured 420 (H) × 300 (W) pixels at 72 dpi subtending 10.6° × 7.6° visual angle at 60 cm distance. Based on an online pilot test of all the trials, we created three sets of equal difficulty (akin to the MFMT) comprising 32 trials each (16 matched trials and 16 mismatched trials per set: sets A, B, and C) with an equal gender split. As with the procedure in experiment 1, we created three versions of all face pairs: colour, grayscale, and mixed.

#### Procedure and apparatus

The procedure and apparatus were identical to experiment 1, except that the viewing distance was fixed at 60 cm (without a chin rest). Participants saw all 96 trials in a random order and the colour condition was counterbalanced for each set.

### Results

No participants were excluded from the analyses. Means and standard deviations across conditions are presented in Table 2*.*

**Table 2:** Average performance for all conditions (standard deviations are in parentheses) in experiment 2

Condition	“Match” accuracy (%)	“Mismatch” accuracy (%)	Overall accuracy (%)	Sensitivity (*d’*)	Criterion (*c*)
Colour	74.5 (11.8)	78.2 (15.1)	76.3 (10.6)	1.58 (0.72)	0.08 (0.31)
Grayscale	67.8 (14.8)	77.9 (17.2)	72.8 (11.1)	1.41 (0.70)	0.19 (0.43)
Mixed	78.2 (14.1)	68.1 (19.1)	73.1 (10.1)	1.41 (0.73)	- 0.16 (0.42)

Percentage correct was analysed for matched trials, using one-way within-subjects ANOVA with three levels (colour, grayscale, and mixed). There was a significant main effect of image hue, *F*(2,102) = 11.61, *p* < .001, η^2^_p_ = 0.18. Follow-up pairwise comparisons (Bonferroni corrected) showed that, as in experiment 1, participants were more accurate in the colour and mixed conditions than in the grayscale condition, *p* < .001, *d* = 0.51 (95% CI 0.22, 0.82) and *p* < .004, *d* = 0.73 (0.41, 1.10) respectively (see Table 2 for a summary of means and SDs). Again, as in experiment 1, the mixed and colour conditions did not differ from each other, *p* = .36, *d* = 0.29 (95% CI − 0.05, 0.68).

Accuracy was also examined for mismatched trials, using within-subjects ANOVA with three hue levels. The results once again followed those of experiment 1. There was a significant main effect of condition, *F*(2,102) = 15.32, *p* < .001, η^2^_p_ = 0.23. Pairwise comparisons (Bonferroni corrected) revealed that the accuracy was lower in the “mixed” condition than in colour and grayscale conditions, *p* < .001, *d* = 0.59 (95% CI 0.37, 0.86) and *p* < .001, *d* = 0.55 (95% CI 0.29, 0.83), respectively. Accuracy in grayscale and colour conditions did not differ, *p* = 1, *d* = .0.02 (95% CI − 0.23, 0.27).

**Fig. 4:**
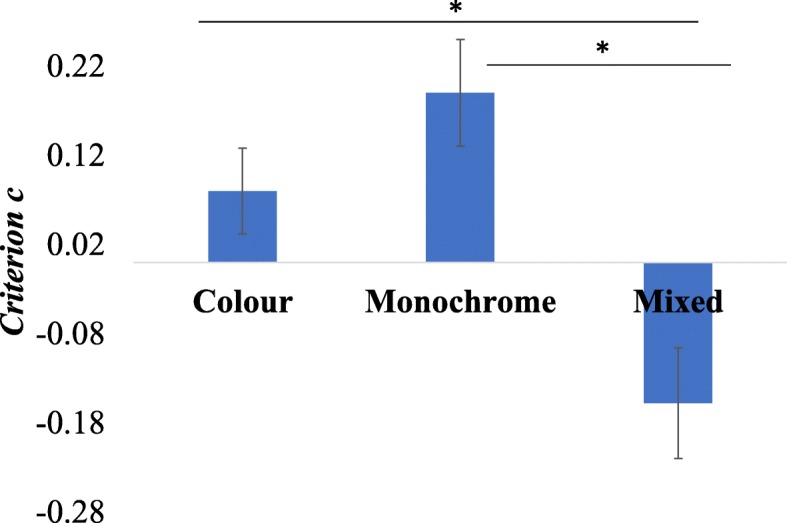
Differences in response bias across three hue conditions in experiment 2. Error bars represent the SEM

We again calculated *d* prime and criterion *c* for all participants. One-way ANOVA of *d* prime scores was non-significant, *F*(2,102) = 1.61, *p* = .204, η^2^_p_ = 0.031. Similarly to experiment 1; we only conducted the follow-up analyses on *d* prime for the critical comparison between the colour and the mixed conditions, *t*(51) = 1.59, *p* = .12, *d* = 0.22, (95% CI− 0.06, 0.49). Participants showed significantly different response bias depending on the condition, *F*(2,102) = 24.65, *p* < .001, η^2^_p_ = 0.33. Follow-up comparisons (Bonferroni corrected) showed that participants had a significantly less conservative bias in the mixed condition than in the colour condition: *p* < .001, *d* = 0.66 (95% CI 0.38, 0.97), and grayscale conditions *p* < .001, *d* = 0.82 (95% CI 0.56, 1.10). The difference in the bias between the grayscale and the colour conditions was not significant after Bonferroni correction, *p* = .093, *d =* 0.29 (95% CI 0.02, 0.55) but note the moderate effect size (see Fig. 4.).

### Pooled effect sizes from two experiments

To increase the statistical power of our analyses, we conducted a meta-analysis of the effect sizes for differences in sensitivity (*d* prime) and criterion *(c)* between the two critical conditions, i.e., colour and grayscale. This allowed us to compute pooled effect sizes for 94 participants. The pooled effect size (*d+*) for sensitivity (Hedges-Olkin method conducted in StatsDirect software) was small: *d+* = 0.28, (95% CI − 0.01, 0.56). An analogous analysis of the bias yielded a medium pooled effect size: *d+* = 0.61 (95% CI 0.32, 0.91).

### Discussion

Experiment 2 replicates the difference in response bias first observed in experiment 1 using a new, more ecologically valid and well-matched face set. Again, participants showed a more conservative response bias (they were more likely to reject a pair as mismatch) in the colour and monochrome conditions than in the mixed condition. Unlike in experiment 1, where in the mixed condition there was no difference in accuracy between matched and mismatched trials, here participants were less accurate in their responses to mismatched trials than matched trials by 10.1%. Put simply, participants were more likely to respond “match” in the mixed condition.

Although the difference between sensitivity scores in the colour and mixed conditions was not formally significant, the effect size (*d*) was within the confidence interval range indicated in experiment 1, providing additional evidence for an effect, albeit somewhat smaller. In sum, these results further support the detrimental effect of hue incongruency on face-matching performance (Additional file 2).

## General discussion

In two experiments, we examined how well people can discriminate whether two images show the same person or two different people using a well-established MFMT (Dowsett & Burton, 2015) (experiment 1) and a new matching test where we included female faces to increase ecological validity (experiment 2). In both experiments, we examined the effect of three image hue conditions: the colour condition where both images were shown in colour, the grayscale condition where both images were shown in grayscale, and the mixed condition where one image of each pair was displayed in colour and the other in grayscale. Analyses of sensitivity revealed a near-significant difference between conditions in experiment 1, where people appeared least able to discriminate pairs in the mixed condition. There was no difference in sensitivity in experiment 2. Critically, there were significant differences in accuracy for matched and mismatched trials across conditions, reflected in different response biases for hue-congruent (colour and grayscale) and hue-incongruent (mixed) pairs. In experiment 1, participants were most likely to respond differently in the grayscale condition, in comparison with the colour and mixed conditions. Additionally, participants were more likely to classify an image pair as a mismatch in the colour condition, in comparison to the mixed condition. Although in comparison to hue-congruent face pairs, the relative accuracy for match and mismatch trials was higher and lower, respectively, the absolute bias in the mixed condition was near zero.

In experiment 2 we sought to replicate this effect using a new set of faces and a new participant sample. Like our experiment 1 results, we found that participants were more likely to classify a pair as a match in the mixed condition, and as a mismatch in the colour and grayscale conditions.

### Colour congruency in face matching

We hypothesised that if colour information facilitates low-level analysis, performance should be reduced in both grayscale and mixed trials relative to colour trials. However, if colour acts as a general diagnostic cue that individuals can use to extrapolate to other examples of the same faces, performance should be unaffected in mixed trials where natural colouration is always preserved in one image. We found limited support for these hypotheses. In matched trials in congruent conditions, participants struggled to recognise faces “together”, and accuracy in the colour and grayscale conditions was lower than in the mixed trials It is possible that colour incongruency disguises subtle differences between faces that may otherwise be picked up on and impairs piecemeal processing. This pattern was reversed in mismatched trials where accuracy was higher in the colour and grayscale conditions than in the mixed trials. If participants were not able to process faces in a piecemeal manner, any perceived “global” differences could have been attributed to the different appearance of the photographs, rather than different identities.

These findings partially support Kramer and Ritchie’s (2016) conclusions where accuracy in their mixed or incongruent condition (e.g., presence of glasses in one of the images) was reduced relative to congruent trials, and participants were more likely to label a pair of images as a mismatch. Here, we also found that young adults are somewhat less accurate in the mixed condition than in the colour condition. Although the effect sizes for this comparison in both experiments are small, they translate to approximately 3% drop in performance when the two images are presented in different colours. Given that in experimental studies, the proportion of mismatched trials is typically 50%, which is presumably higher than the rate of circulating fraudulent ID documents and that infrequent mismatched trials are likely to be missed (Papesh & Goldinger, 2014), it is possible that the drop in performance may be even higher in real-world conditions.

In contrast with Kramer and Ritchie (2016), participants were unbiased in their responses in the mixed condition in a set of male face pairs but biased to respond “match” in a set of male and female face pairs. Kramer and Ritchie’s (2016) task manipulated the appearance of the faces in that identities in the images wore glasses or not, but the photographs were not doctored. Here, we did not deliberately choose photographs in which the appearance of the identities varied by wearing paraphernalia or sporting facial hair. The only manipulation we performed was changing the hue of the images. These differences between Kramer and Ritchie’s (2016) work and ours may result in different strategies adopted by participants. For example, while keeping the colour constant, subtle differences in facial appearance may serve as a cue to reject a pair as a mismatch. However, adding incongruency in image properties (i.e. having one image in colour and one in grayscale) may cause participants to dismiss differences in facial appearance as being due to differences in image properties, thereby making them more likely to say that two images are a match.

Put simply, when the hue of images differs, participants have more factors to consider when attempting to discriminate whether two images show the same person or two different people. Specifically, when both photographs are in grayscale or in colour, individuals may primarily base their decisions on identity-specific factors, i.e. when two images look sufficiently different, there are no other “environmental” variables to consider and a pair is classified as mismatched. However, given that grayscale and colour images are inherently different, participants may inadvertently assume that a perceived difference in the two photographs is either due to the actual identities being different, or to the same person looking very different due to incongruency in hue between the photographs.

Furthermore, eye colour has recently been found to be an important diagnostic feature in face recognition (Abudarham & Yovel, 2016). If eye colour is, indeed, diagnostic, then the difference in hue between grayscale and colour photographs could make it impossible to access this information and lead participants to make decisions based on less diagnostic features, such as eye shape or jaw line. It is possible that this contributed to the low performance in mismatched trials and the relatively low accuracy of grayscale matched trials in both experiments.

These results are also in line with the bias reported by Megreya and Burton (2008) where the authors compared performance between matching of two grayscale photographs and of a person to a grayscale photograph (experiment 3). Participants were more likely to respond “match” in the live condition than in the image condition. Critically, the image-to-image comparisons were always colour congruent (grayscale), but in the live condition grayscale photographs were compared to human actors, a setting similar to our mixed trials. A study utilising a real-life paradigm with photographs of different hue would help to clarify this. A systematic investigation of low-prevalence mismatches for colour and grayscale photographs and a more ecologically valid “live” condition would be a timely addition to this preliminary evidence reported in our two studies (c.f., Calic, 2013).

The critical finding here is that the lowest performance in mismatched trials was in the mixed condition. This has important implications for the use of grayscale images in photographic ID. Given that some official IDs are still produced using grayscale photographs, it is plausible that the level of fraud using such documents may be higher.

### Re-thinking photographic ID and future work

Numerous studies to date have shown that facial image comparison is a difficult task. Even under optimal conditions where two images have the same hue, are taken on the same day, and participants are under no pressure to perform fast and accurately, the error rate is approximately 20% (Burton et al., 2010). This is further affected by change in illumination (Braje, Kersten, Tarr, & Troje, 1998), expression (Chen, Lander, & Liu, 2011; Mileva & Burton, 2018) and time passing between when photographs were taken (Megreya, Sandford, & Burton, 2013). This study adds to the body of evidence that photographic ID is relatively unreliable as a tool for accurate identity verification.

The EU requirements for photographic driving licences state that images must be taken without occlusions and with a plain background, up to 6 months prior to applying for the document. However, although submitted in colour, these photographs are later converted to grayscale. Similar policies apply to Canadian and Polish passports, where images are printed in grayscale (see Fig. 1 for an example of such images). We recommend a systematic investigation into the effect these grayscale IDs have in professional settings, as the risk posed by poorly designed documents is high. The investigations should concentrate efforts not only on security personnel, such as passport and police officers, but also on other staff engaged in identity checks such as airline and security personnel or cashiers.

With respect to ID colour, recent research has shown that forensic examiners (White et al., 2015) and super-recognisers (Phillips et al., 2018) are considerably better at face matching than untrained individuals. Future work should examine the susceptibility of those groups to image colour incongruence.

### Limitations and conclusion

One limitation of our study was that participants were untrained young adults without prior substantial experience in face matching. Those who perform face matching as a part of their everyday job may be more familiar with the limitations of photographic ID and thus more vigilant in detecting attempts at fraud. Nonetheless, young adults often work in retail where they may engage in selling age-restricted items (such as alcohol or tobacco) so it was important that we investigate performance in this population.

In sum, we showed that image colour incongruence can contribute to naive participants accepting a mismatched pair of images as the same person. This finding has considerable implications for the design of photographic ID where grayscale photographs are often used. While it is unclear what effect such inconsistency has on trained or highly skilled individuals, we urge the policy makers to re-think image colouration in photographic identity cards and incorporate this limitation into training for staff for whom ID checks are a part of their everyday job.

## Additional files

Additional file 1: Data colour match experiment (Exp.) 1 (SAV 6 kb)

Additional file 2: Data colour match Exp. 2 (SAV 13 kb)

## Data Availability

SPSS data files are included in the supplementary materials.
